# 6.P. Skills building seminar: Children and young people: engaging the unheard stakeholder

**DOI:** 10.1093/eurpub/ckac129.398

**Published:** 2022-10-25

**Authors:** Organised by: EUPHA-CAPH, EUPHAnxt, Chair persons: Hellen Skirrow (UK), Jinane Ghattas (EUPHAnxt)

## Abstract

Global shocks from the COVID 19 pandemic have disproportionately impacted children and young people (CYP) and their families. Underlying health disparities have widened as a result of disruptions to healthcare, education and economic impacts. There are compelling arguments to engage children and young people in rebuilding society and systems from emerging public health threats including the covid 19 pandemic, the climate crisis and conflicts. Children and young people like others in society have a right to be involved in decisions that impact their lives and health but are too often unheard. Engaging those affected most by health policy ensures relevance and can improve adoption of interventions. Involving children and young people in strategic decision making can also improve their citizenship skills and improve health literacy. Although many children and young people have contributed to debates and publicly demonstrated their collective views on a variety of issues such as climate change, movements including #timesup, #Blacklivesmatter and #Metoo, their voices are seldom heard in policy and decision making arenas. In 2021, EUPHA CAPH and EUPHANxt sections collaborated to host the inaugural Engaging the Unheard Stakeholder workshop, supporting 2 young people aged <25 to attend the EPH and lead the workshop. (links). CAPH is now committed to make this a regular feature as part of our action plan to support young voices in public health.

**Learning outcomes:**

• Develop insight into innovative ways to support and engage young people in public health policy making.

• Practise advocacy skills to facilitate young people's voices in research & policy

**Activities:**

Participants will hear from young people who are championing activism or have been involved in shaping public policy.

Interactive skills building breakout session involving role play and scenarios to challenge their unconscious bias and learn skills as ‘active bystanders’ to empower young people

**Key messages:**

• Despite major movements where children and young people have demonstrated their views on a variety of issues such as climate change, their voices are seldom heard in policy and decision making arenas.

• Public health professionals can advocate, support and engage children and young people in public health policy making but lack awareness and skills in advocacy to do this effectively.

